# Studying friction while playing the violin: exploring the stick–slip phenomenon

**DOI:** 10.3762/bjnano.8.16

**Published:** 2017-01-16

**Authors:** Santiago Casado

**Affiliations:** 1 Madrid Institute for Advanced Studies in Nanoscience (IMDEA Nanoscience), Faraday 9, Ciudad Universitaria de Cantoblanco, 28049 Madrid, Spain

**Keywords:** atomic force microscopy, bow hair, friction, stick–slip, tribology, violin

## Abstract

Controlling the stick–slip friction phenomenon is of major importance for many familiar situations. This effect originates from the periodic rupture of junctions created between two rubbing surfaces due to the increasing shear stress at the interface. It is ultimately responsible for the behavior of many braking systems, earthquakes, and unpleasant squeaky sounds caused by the scratching of two surfaces. In the case of a musical bow-stringed instrument, stick–slip is controlled in order to provide well-tuned notes at different intensities. A trained ear is able to distinguish slight sound variations caused by small friction differences. Hence, a violin can be regarded as a perfect benchmark to explore the stick–slip effect at the mesoscale. Two violin bow hairs were studied, a natural horse tail used in a professional philharmonic orchestra, and a synthetic one used with a violin for beginners. Atomic force microscopy characterization revealed clear differences when comparing the surfaces of both bow hairs, suggesting that a structure having peaks and a roughness similar to that of the string to which both bow hairs rubbed permits a better control of the stick–slip phenomenon.

## Introduction

Friction is generally understood to be the resistance to the sliding motion of objects. It is an everyday life phenomenon that originates from the atomic-scale asperities found at the interfacial contacts [1]. However, substantially different physical descriptions apply when friction is studied at the nanoscale compared to the macroscale, indicating that more exploration at the mesoscale is needed to bridge the models from the two regimes [2–3]. Amongst the possible friction effects, the stick–slip regime is present in many familiar cases. For instance, it influences the behavior observed in many braking systems, earthquakes, the squeaky sounds caused by the scratching of two surfaces like a chalk on a blackboard, the grinding of a rusty hinge, or the wear of articular joints [4]. This phenomenon is caused by the rupture of equilibrium occurring when two materials are steadily being rubbed against each other, producing a continuous and periodic fluctuation between static and dynamic regimes [5–6].

Commonly analyzed from a theoretical perspective, an experimental study of the stick–slip regime at the mesoscale presents important practical difficulties and challenges. For example, a design with a wide flat area would yield limited or confusing information about the evolving shape at the interface during measurements. Random changes on the surface geometry depend on the initial height variation, material heterogeneities, or plastic deformations [7], but it is very difficult to control them during the experiment. It is therefore desirable to minimize these possible sources of systematic errors or complications by reducing the contact region of the rubbing pieces without decreasing the signal-to-noise ratio of the relevant parameter, and by limiting the analysis to interfaces that can endure many friction cycles without being altered.

**Figure 1:**
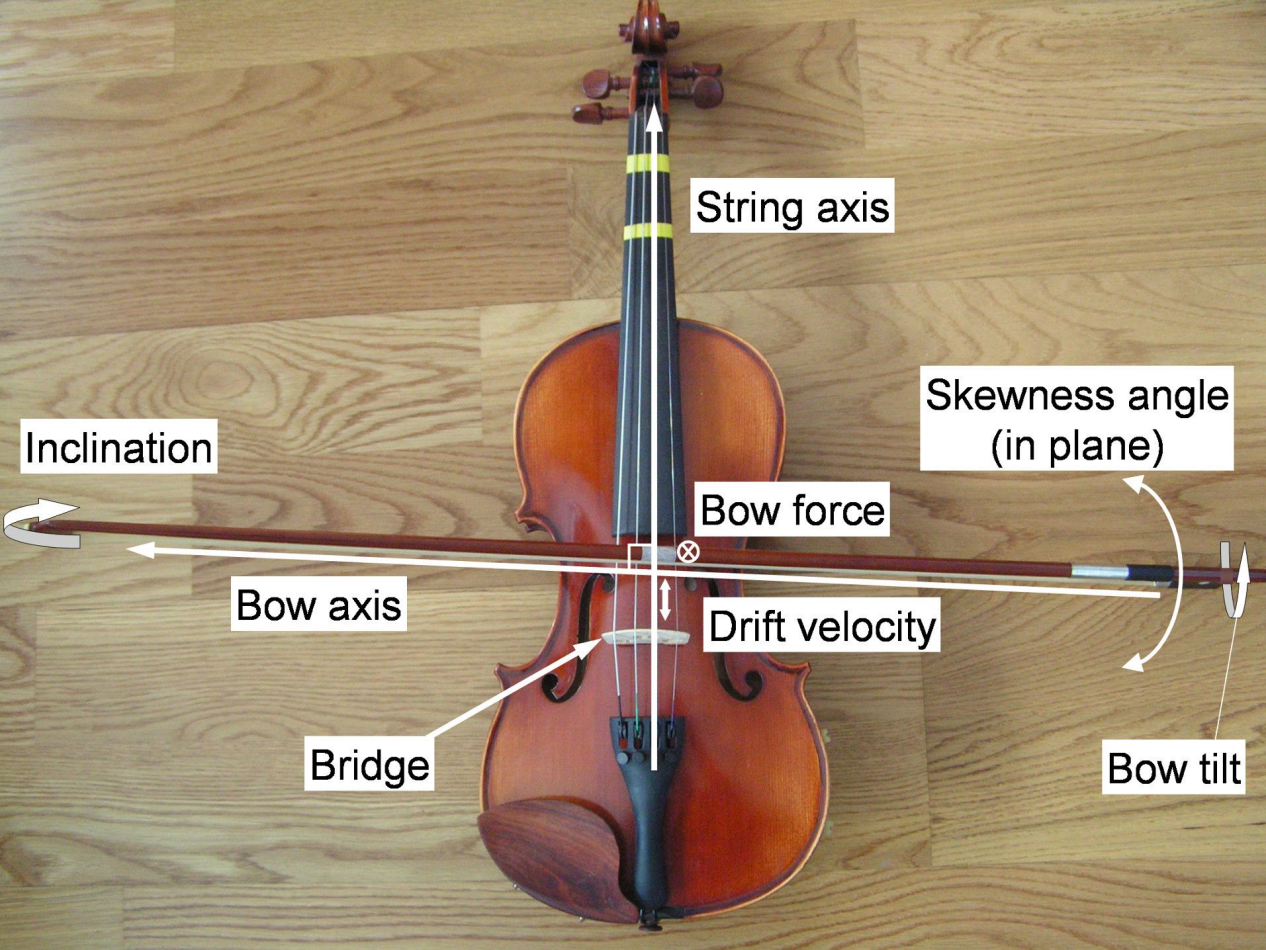
Scheme of the different parameters involved in violin performance.

Fortunately, there exist common tools that have both features, making them exceptional platforms to study the static–dynamic transition. This is the case of bow-stringed musical instruments, where the stick–slip effect is controlled to produce a wide variety of well-tuned sounds. A violin, for example, can produce musical notes whose frequency and intensity (brilliance) can be varied as required during the performance by rubbing a bundle of fibers against metal strings [8]. The contact surface is then reduced to the touching points of perpendicular cylinders, where friction occurs on the same materials all along the bow length, and the process is repeated many times without disturbing the emerging sound. Previous studies have shown that only a few mechanical parameters determine this tune: skewness angle, maximum bow velocity, drift velocity (or bow–bridge distance dependence), bow position, tilt, inclination, and bow force [9–11] (see Figure 1). Therefore, if these parameters are fixed, we can use the resulting sound of a bow-stringed musical instrument as a signal to measure characteristics of the mesoscale friction between the bow hairs and the strings.

However, very few studies in the literature are concerned with the nanoscopic analysis of the bow hairs or the strings of musical instruments. In the present work, an optical microscope and an atomic force microscope (AFM) are used to inspect the structural differences at the micro- and nanoscale of two types of bow hairs. One is a natural horse tail hair bow used in a philharmonic orchestra, which permits a good control of the tune and the brilliance of the sound (denoted as sample 1 hereafter); and the other one is a synthetic fiber not able to generate such a fine control, independent of the experience, skill, or the anatomical characteristics of the particular player during the performance (denoted as sample 2). The latter has appeared on the market only recently, offered at a competitive price. A comparison between surface characterizations provides an explanation of the different acoustic outputs and proposes a way to experimentally explore the stick–slip phenomenon emergence, taking advantage of the scientific and practical efforts already made concerning the violin design.

## Results and Discussion

**Figure 2:**
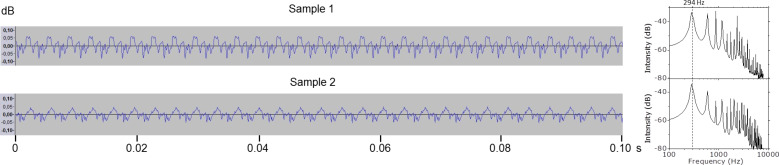
Sound waves and Fourier analyses of samples 1 and 2 with rosin particles.

**Figure 3:**
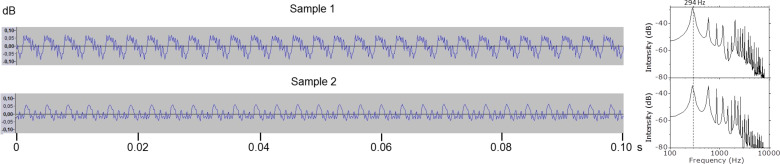
Sound waves and Fourier analyses of samples 1 and 2 after cleaning.

The sound intensity was plotted versus time in Figure 2 for samples 1 and 2 in the presence of rosin particles, and after being cleaned in Figure 3. Although cleaning may also extract natural lipids from the hair surface, the periodicity of the highest peaks is maintained constant throughout the four cases. The Fourier analyses, also included in the same figures, confirm that all exhibit a main peak at around 294 Hz, the frequency corresponding to the D_4_ note. However, when comparing the intensity distribution of each sound wave along a single period, clear differences appear between the two samples. Only when they have rosin particles both look similar, but, after cleaning, although they achieve an analogous upper intensity, the intensity of the signal produced with sample 2 decays quickly without forming a valley, whilst with sample 1 it decreases gradually and a deep valley is formed. Since this is related to the stick–slip regime produced by the friction of each sample with the same D-string, these variations in the intensity distribution of the sound waves can be attributed to the surface roughness of the two samples.

**Figure 4:**
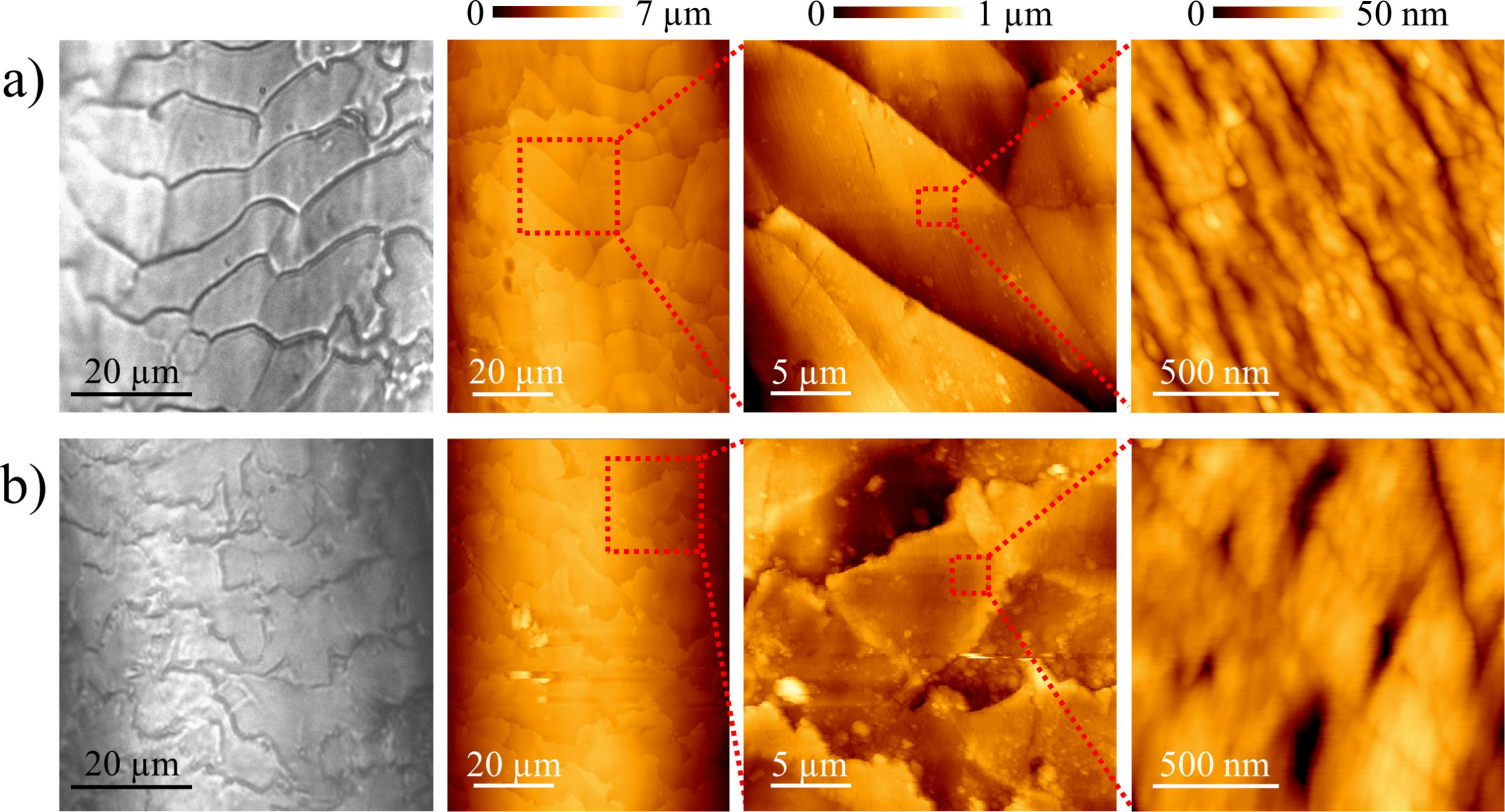
Images of sample 1 (a) and 2 (b) cleaned surfaces, showing geometrical differences at various scales.

**Figure 5:**
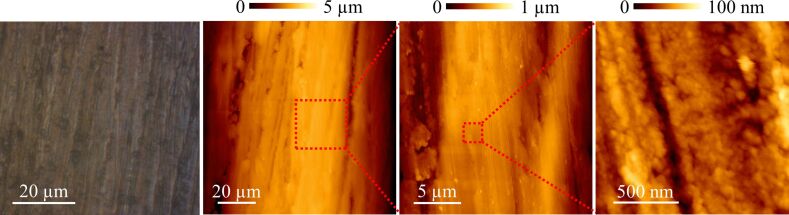
Surface characterization of the D-string of a violin.

Structural differences between the surfaces of both samples can be checked by an optical microscope and an AFM. In Figure 4, images of the samples taken at the same magnifications can be compared. Although some differences between the samples are made apparent from the optical microscope images (first column of each row), it is at the micrometer scale (middle columns) and at the nanoscale (last column) where structure variations are more evident. The two samples present a scaly appearance, with flake widths of tens of micrometers for sample 1 and smaller and more breakable ones in sample 2. Since it is the friction against the D-string what is under study, a geometrical characterization of the D-string topography is also required. This is shown in Figure 5, where the same magnifications as in Figure 4 were selected. Other violin strings were also inspected, but they all presented similar surface characteristics at these scales. Hence, only a representative D-string was analyzed in detail.

**Figure 6:**
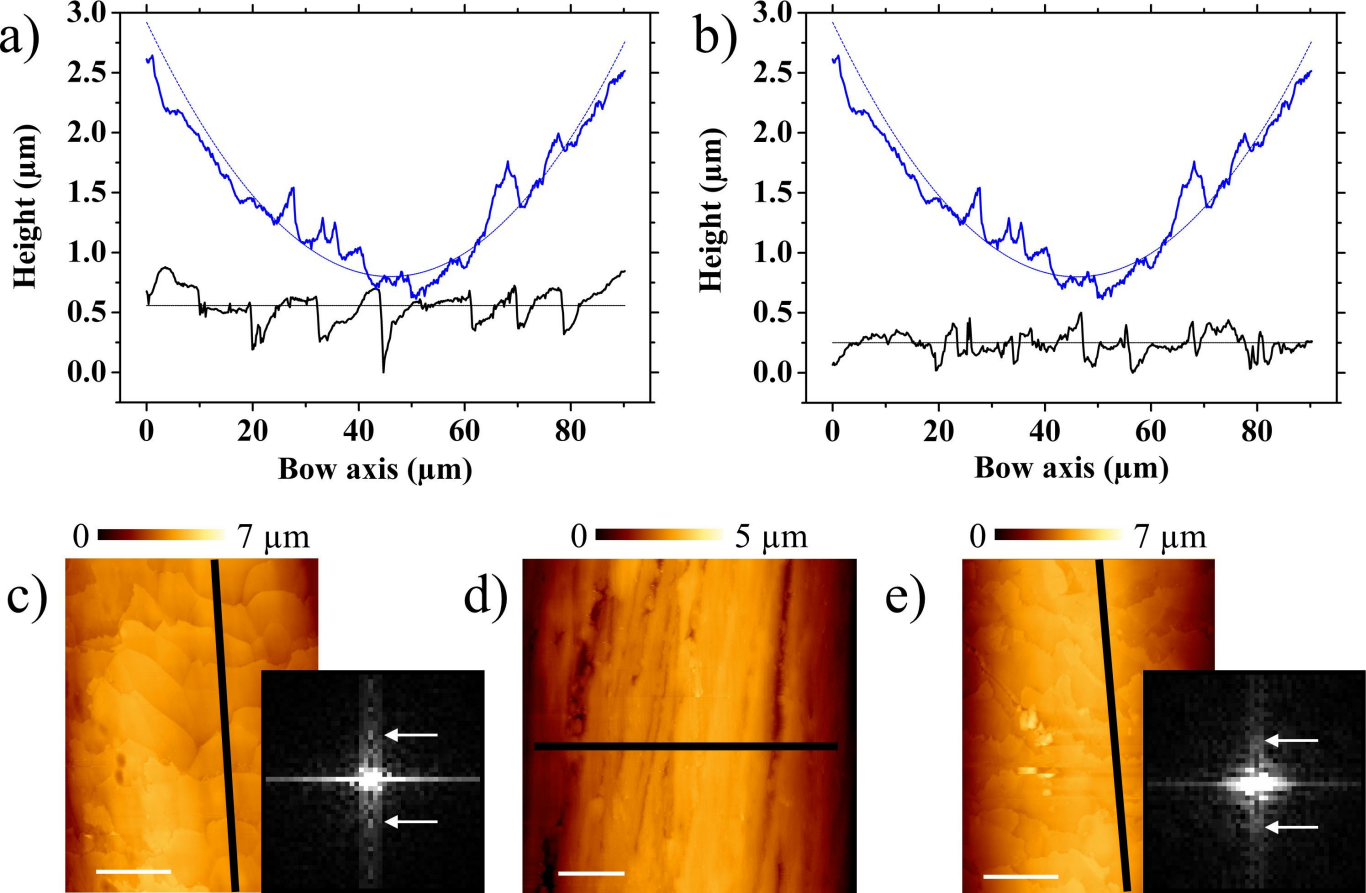
Line profile comparison between D-string (upper plots, blue line) and sample 1 (a) and 2 (b) surfaces (black lines). The horizontal lines are the average of the hair profiles, and the curved lines correspond to the section of a cylinder of the D-string diameter. (c–e) Profile extraction lines for sample 1 (c), the D-string (d), and sample 2 (e). Insets in pictures c and e are the 0.5 × 0.5 µm^−1^ central areas of the corresponding two-dimensional fast Fourier transforms at the same intensity contrast. White arrows indicate frequencies associated with 9 µm vertical wavelengths in both cases. All white scale bars are 20 µm.

In Figure 6, line profiles obtained from AFM characterization of samples 1 (Figure 6a) and 2 (Figure 6b) are compared to a perpendicular profile of the D-string (upper plots). Sample 1 displays a periodicity and roughness not observed in sample 2, in agreement with the corresponding two-dimensional fast Fourier transforms. Despite their moderate sharpness due to irregularities, the frequencies corresponding to wavelengths around 9 µm along the bow axis appear more blurred in the second case. These morphological asymmetries at the microscale may cause the distinct friction control at the macroscale. Clearly, a rough surface like that shown in sample 1 can tear stronger from the string, provided the hair flakes remain unbreakable during the whole process, and store more potential energy from the string bending in the static friction regime. When this energy is released, the slipping motion should not be stopped until the string velocity is significantly reduced. A profile like the one corresponding to sample 2 may prevent this movement in the dynamic friction range. This is indeed similar to that observed in the sound waves recorded from clean fibers in Figure 3: a peak is achieved, but quickly diminishes to a flat region. Instead, sample 1 shows a more regular saw tooth profile and a deeper valley, confirming the assumption. To ascertain if the roughness enhancement of the basic structure is the only feature responsible for the different stick–slip phenomenon control, the same structures were analyzed in the presence of rosin particles.

**Figure 7:**
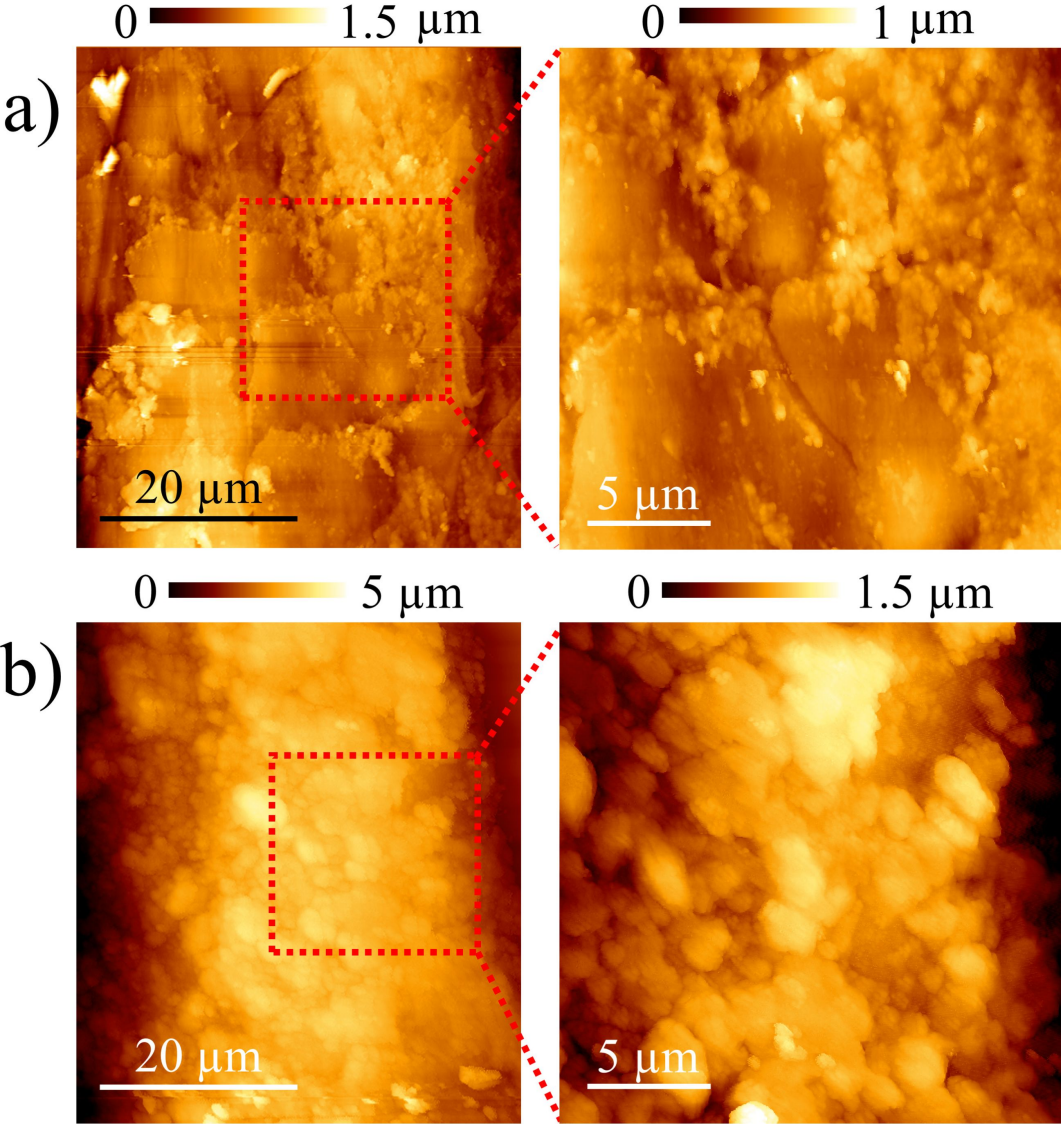
Sample 1 (a) and 2 (b) surfaces covered by rosin particles, in the same conditions employed during the last performance.

Figure 7 compares samples 1 and 2 presenting exactly the same conditions employed during the last performance. The results clearly show that sample 2 requires much more rosin particles than sample 1. When using synthetic bow hairs, it is known from experience that they have to be covered by more rosin material, requiring reiterative application of coatings as well during the performance. More frequent squeaky sounds and a decrease in the intensity using sample 2 indicate that the static friction capacity is reduced and that spurious sticking phases appear during the slipping range. The application of more rosin particles all along the bow hairs recovers the original sound and makes it similar to that produced by sample 1, as compared in Figure 2.

**Figure 8:**
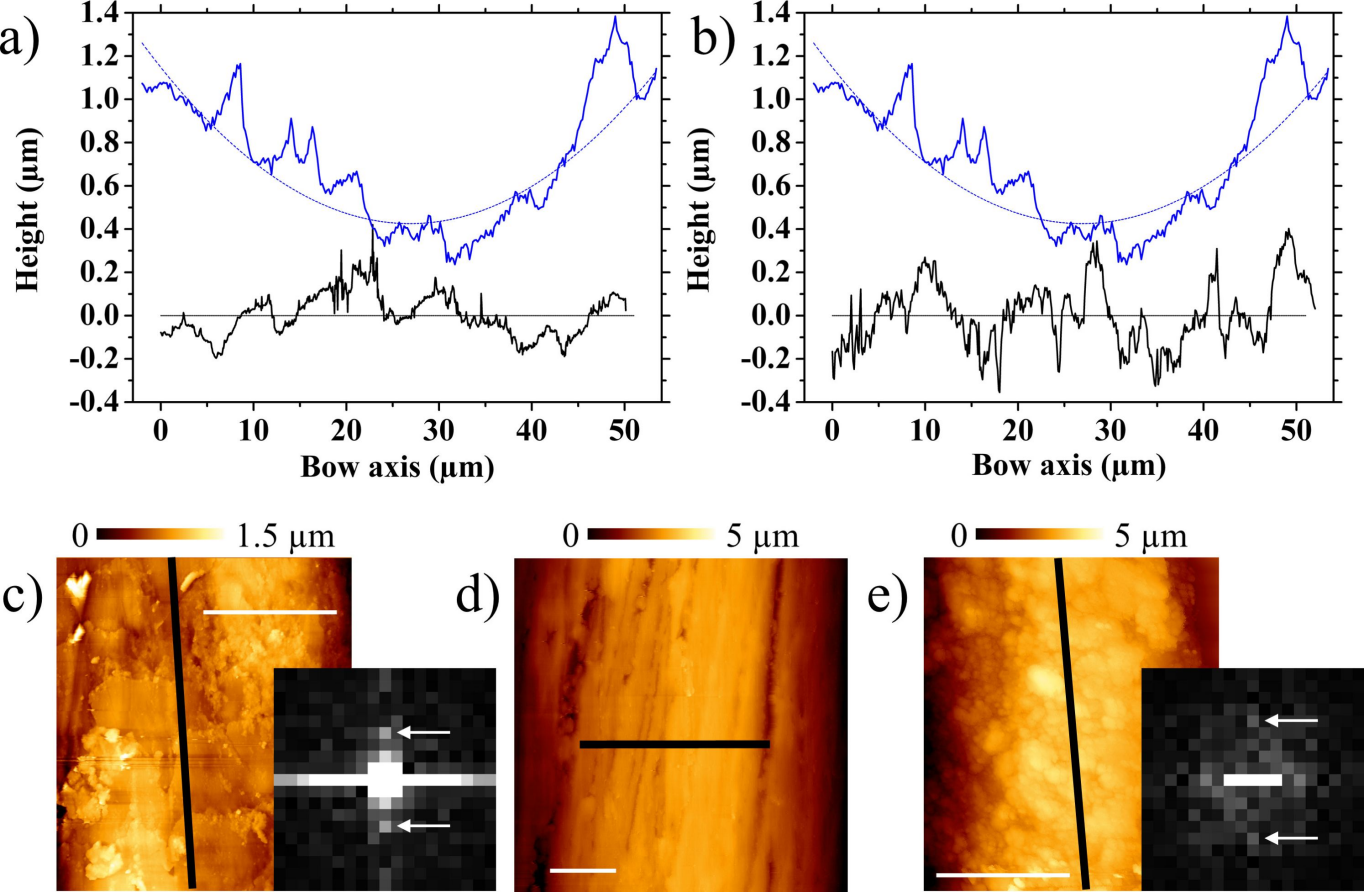
Comparison between the same D-string (upper plots, blue lines) profile shown in Figure 6 and the profiles of sample 1 (a) and 2 (b) surfaces covered by rosin particles. The curved lines correspond to the same section of the cylinder depicted in Figure 6. (c–e) Profile extraction lines for sample 1 (c), the D-string (d), and sample 2 (e). Insets in c and e are the 0.5 × 0.5 µm^−1^ central areas of the corresponding two-dimensional fast Fourier transforms at the same intensity contrast. White arrows indicate frequencies associated with 9 µm (c) and 7 µm (e) vertical wavelengths. All white scale bars are 20 µm.

Figure 8 depicts profile differences between the two samples covered with rosin, contrasted to the perpendicular D-string profile (upper plots). Although the increase of irregularities due to these particles blurs both two-dimensional Fourier transforms, the frequencies associated with 9 µm wavelengths along bow axis of sample 1 are still present, whilst others around 7 µm appear in sample 2. The presence of more rosin particles optimizes the roughness of the sample 2 to enhance the stick–slip phenomenon, but the resultant is a fragile surface. This loose coating is rearranged during the fiddle and many particles are lost, explaining the requisite of continuously adding more rosin during the performance, and the cleaning after it.

The comparison between the surface roughness of these two bow hairs at the mesoscale have proven that, for controlling the stick–slip phenomenon, a wedged hard structure is desirable. Roughness at this scale helps to longer maintain the sticking phase and to store more energy, without prohibiting the sliding phase. According to the Helmholtz model [12], during the static range the bow hairs force the string to bend, forming a corner (kink peak) at the pulling position. This situation persists until the maximum static friction is reached. At this moment, the string eventually loses the grip of the bow and all the stored energy is released, starting the slipping phase. Whilst the perturbation corner travels along the string producing the sound, dynamic friction is maintained at the bow–string interface. When the sliding velocity is reduced to a critical value (i.e., when the corner is again close to this interface), static friction is recovered and the bow forced movement feeds the string vibration. The process is repeated as long as the bow velocity is maintained. Corrections to the model focus on the string perturbation corner shape [13], because it affects the timing of the different Helmhotz model phases, modifying the tune. The occurrence of this corner shape not only depends on the elastic characteristics of the string, but on the stick–slip friction with the bow as well. Longer static friction ranges will allow better control of the brilliance of the sound under different bow forces, providing an important expressive mean to the player; but if it exceeds a certain value, the vibration is stopped while sliding and raucous or aperiodic motions appear, corresponding to scratchy sounds. Acceptable sounds arise only at a certain range of static and dynamic friction forces [11]. Furthermore, if the performing skill is appropriate and the stick–slip control is sufficiently enhanced, it is possible to use a particular friction characteristic like the flattening effect [14], related to the stick–slip phase hysteresis, as a mode of expression during the fiddle.

Consequently, the more regular sound wave intensity distribution and the better adjusted tune obtained in sample 1 indicate that, in this case, static friction is enhanced and dynamic friction is reduced, as compared to sample 2. The analysis of the surfaces covered by rosin particles confirmed that a structure characterized by distinct peaks contributes to a better control of the stick–slip friction phenomenon. Moreover, studies characterizing new and old bow hairs point towards the same conclusion [15], justifying the habit of changing the hairs of the bow regularly.

Although a detailed experimental analysis of the nominal and real areas of contact may yield quantitative data to be studied using friction theories [16–18], some features prevent this exploration. Firstly, these theories concern two flat surfaces and not two cylindrical shaped materials presenting different pressure distribution when a normal load is applied. Secondly, the sticking of the string is produced on diverse points by the cooperative effect of many individual hairs. And finally, torsion modes of the string must be also included in the theory. Nevertheless, it is possible to estimate the roughness differences measured by comparing the standard deviations of the profiles shown in Figure 6 and Figure 8, along 90 µm and 50 µm, respectively. Thus, it is 142 nm for (cleaned) sample 1 and 92 nm for (cleaned) sample 2, and changing to 151 nm when coated with rosin particles. Interestingly, the standard deviation of the string profile shown in the same figures, relative to the perfect cylindrical shape also plotted, was 148 nm. This is of the same order of those cases where the tune was enhanced, i.e., where the stick–slip effect was better controlled. Hence, the roughness responsible for the stick–slip occurrence in this scenario seems to be in the tenth of a micrometer to a micrometer range. Therefore, only those topographical structures observed when characterizing regions tens of micrometers wide are directly involved. The nanostructure differences depicted in Figure 4 may also play a role, but likely related to the ability to store rosin particles.

## Conclusion

The ultimate causes from which the stick–slip friction phenomenon emerges at the mesoscale is still under debate, mainly through model systems. To gain insight into this matter, surface characterization of devices with a fine control of a measurable physical effect triggered by friction at this scale is crucial. Optical and AFM analysis on two different bow hairs used with a violin proved that a surface characterized by periodic peaks and a rough microstructure of the same order of the rubbing string roughness favors the control of the fiddle, i.e., enhances the stick–slip range at the interface. The characterization of the surfaces covered by rosin particles, in the actual conditions used during the last performance, confirmed the statement. Further experiments using artificial hairs with different geometries of regular shapes, setting the skewness angle perpendicular and controlling bow velocity and bow force on the string, can provide valuable information regarding friction dependence on the roughness at the mesoscale. These experiments will have the advantage of testing minimized interfaces checking clear macroscale sound effects, and comparing them with results obtained from modelling.

Understanding the best bow hair structure can also encourage a better synthetic construction. Currently, most of the advanced bows are still made of breakable natural horse hair, and the first attempts to produce synthetic bow hairs are not optimized yet. An improvement in this field could eventually yield to a fiber made of a stronger material (not requiring frequent replacement) that avoids the use of rosin particles, but preserves (or improves) the sound quality obtained using natural horse tail hairs.

## Experimental

Two bow hairs were compared: a natural horse tail hair, used in a professional philharmonic orchestra, and a synthetic hair used with a Bestler violin for beginners. Both hairs were tested under optimal musical conditions in all the possible violin scales, checked and recorded by the same professional violin player, on the same violin, and on the same string (D). The age of the two samples was approximately the same. Therefore, no effect except their surface difference is the responsible for their sound variation. The recorded sounds were analyzed using Audacity software.

In order to check their surface characteristics, atomic force microscopy images were taken on both surfaces. A JPK Nanowizard II AFM was employed, which was coupled to a Ti-U Nikon inverted optical microscope. Pieces of samples 1 and 2 were measured before and after being cleaned by 5 min of ultrasonic bath immersed in acetone. Fastened along an optical microscope glass slide using scotch tape on both sides, the samples proved to be suitably attached for AFM measurement purposes. The coupling to the optical microscope significantly helped in the localization of the upper hair region. The surface characterization of one standard string (Synthetic/Silver Mittel Envelope D-string, Pirastro Tonica) was also measured using the same protocol. All AFM characterizations were made in air and ambient conditions, operating in dynamic mode. NT-MDT NSG01 cantilevers of around 150 kHz and 5 N/m, and tips of 6 nm typical radius, were used. Two-dimensional Fourier transforms were obtained using Gwyddion software.

The samples were measured and sounds were recorded before and after cleaning as during the performance, rosin particles are also present [19]. Usually made from pine resin and molded in solid blocks, the rosin material must be applied along the bow hairs before starting the fiddle. Experience has demonstrated that the amount of these rosin particles should lie in a precise range. Too many can yield a scratchy sound, and not enough produces frequent squeaky unpleasant noises. The optimum quantity is commonly detected by the player skill and the knowledge of the particular instrument. If real friction conditions are to be related to the surface characteristics of the hair–string interface, an analysis of the surface changes and sound variations produced by these rosin particles is required.
